# Severity as a Priority Setting Criterion: Setting a Challenging Research Agenda

**DOI:** 10.1007/s10728-019-00371-z

**Published:** 2019-05-22

**Authors:** Mathias Barra, Mari Broqvist, Erik Gustavsson, Martin Henriksson, Niklas Juth, Lars Sandman, Carl Tollef Solberg

**Affiliations:** 1 The Health Services Research Unit – HØKH, Akershus University Hospital, Sykehusveien 25, Postboks 1000, 1473 Lørenskog, Norway; 2 Department of Medical and Health Sciences, The National Centre for Priorities in Health, Linköping University, Linköping, Sweden; 3 Department of Culture and Communication, Centre for Applied Ethics, Linköping University, Linköping, Sweden; 4 Division of Health Care Analysis, Department of Medical and Health Sciences, Linköping University, Linköping, Sweden; 5 Department of Medical and Health Sciences, Center for Medical Technology Assessment, Linköping University, Linköping, Sweden; 6 Stockholm Centre for Healthcare Ethics (CHE), LIME, Karolinska Institutet, Solna, Sweden; 7 Global Health Priorities, Department of Global Public Health and Primary Care, Faculty of Medicine, University of Bergen, Bergen, Norway

**Keywords:** Severity, Priority setting, Ethics, Health policy, Guidelines, Research agenda

## Abstract

Priority setting in health care is ubiquitous and health authorities are increasingly recognising the need for priority setting guidelines to ensure efficient, fair, and equitable resource allocation. While cost-effectiveness concerns seem to dominate many policies, the tension between utilitarian and deontological concerns is salient to many, and various severity criteria appear to fill this gap. Severity, then, must be subjected to rigorous ethical and philosophical analysis. Here we first give a brief history of the path to today’s severity criteria in Norway and Sweden. The Scandinavian perspective on severity might be conducive to the international discussion, given its long-standing use as a priority setting criterion, despite having reached rather different conclusions so far. We then argue that severity can be viewed as a multidimensional concept, drawing on accounts of need, urgency, fairness, duty to save lives, and human dignity. Such concerns will often be relative to local mores, and the weighting placed on the various dimensions cannot be expected to be fixed. Thirdly, we present what we think are the most pertinent questions to answer about severity in order to facilitate decision making in the coming years of increased scarcity, and to further the understanding of underlying assumptions and values that go into these decisions. We conclude that severity is poorly understood, and that the topic needs substantial further inquiry; thus we hope this article may set a challenging and important research agenda.

## Introduction

Priority setting in health care is ubiquitous and inevitable. Broadly, priority setting in health care can be understood as any mechanism, formal or informal, which deliberates between the possible uses of available resources. Priority setting takes place from the bed-side-level to decisions at the national level [2, 36, 47, 55, 67, 73, 83, 93, 124].

The literature on *priority setting criteria*—a crucial ingredient of real-world priority setting—and their ethical underpinnings is as rich as it is diverse. Ethicists, health care professionals, economists, and policy makers have all contributed to the debate on suitable principles for priority setting in health care. Arguments from utilitarian, egalitarian, and prioritarian camps, among others, have been advanced but no thorough consensus seems to exist [14, 15, 19, 22, 27, 33, 34, 39, 44, 51, 57, 64, 65, 72, 76, 81, 89, 95, 96, 102, 103, 115, 116, 119–121]. Moreover, the lack of attention to underlying values has given a false impression that there exist value-neutral methods and techniques to inform priority setting [45, 50, 122].

Recent studies investigating values behind policies and methods have disclosed a greater diversity of priority setting principles and criteria across countries than expected [8, 15, 58]. Safety, efficiency, cost, and budgetary constraints together constitute classical cost-effectiveness, which in priority setting ethics is built upon utilitarian considerations. In Norway and Sweden, however, considerable efforts have been made to integrate non-utilitarian ethical principles into the nations’ priority setting policies, and we will briefly recount the work of the past decades below.

A common concern in both Norway and Sweden—and elsewhere—is how to accommodate a perceived obligation to prioritise those with severe conditions. Both general and more specific severity criteria have been suggested and implemented within these nations’ publicly funded health care systems.^1^ Indeed, *severity* has been paramount in the development of official priority setting guidelines and legislation in Norway and Sweden. On the surface, these criteria appear to be fairly uncontroversial and widely accepted as relevant and legitimate. We will argue in this article that severity—*qua* priority setting criteria—is, in fact, controversial when operationalised, ethically ambiguous, and that it is likely that there exist irreconcilable views about when and to what extent a condition is severe. Hence, there is a need for more research within the area of severity. A key ingredient in this research activity on severity will be to highlight the controversies: exactly what is unknown or controversial? The aim of this article is to set a research agenda from the interdisciplinary perspective of scholars engaged in priority setting research, focusing on severity. Although the questions posed below can be seen as mainly philosophical, answering them has implications for other areas, e.g. health economics. Hence, input from these other areas is likely to be fruitful, especially when it comes to concrete issues such as measuring severity. Against this background, the authors represent several different disciplines.

We will argue that the concept of severity can be viewed as a multidimensional concept, drawing up research questions related to concepts like need, urgency, fairness, social consequences, mortality versus morbidity, and human dignity. At the same time, a Scandinavian perspective on severity might be conducive to advancing the international discussion, given its long-standing use as a priority setting criterion in Norway and Sweden. This is despite having reached rather different conclusions—and hence they currently use different formal priority setting criteria (see next section). Since different accounts of severity are, in fact, prevalent in different jurisdictions, these diverse accounts need to be explicitly recognised and deliberated. Otherwise, we believe that the long-term legitimacy of official priority setting policy may be compromised. This concern builds on a literature of the role of the social contract in health care policy, originating with John Rawls and Norman Daniels [20, 21, 86], but also more recent research on the need for legitimacy in priority setting [15, 30, 91, 95, 122]. It may be prudent, therefore, to analyse severity both as an *essentially contested* concept [16, 32], and severity as (at least temporary) a basis for *incompletely theorized agreements* [92, 113]. Considering the framework of essentially contested concepts may serve to highlight that the labelling of a condition as severe may serve as a mechanism though which a claim of higher priority is mediated; suggesting that (sometimes) severity is a relative concept. The theory behind incompletely theorized agreements promises a rich framework for analysing at what level disagreements on definitions of severity occur: Do agents disagree on fundamental ethics and background theories? Do they diverge on mid-level principles for operationalising severity, or is it the classification of specific cases into agreed-upon abstract categories that causes tensions? Severity matters to people, it seems. In this paper we highlight a number of points of disagreement on how and why.

The remainder of this article is structured as follows: first we give a brief history of the current severity criteria in Norway and Sweden. Next, we give a rudimentary taxonomy of the identified ethical principles and concerns forming the basis of the severity criterion in the two countries. Thirdly, we present what we think are the most pertinent questions to answer regarding severity, in order to facilitate decision making in the context of increased scarcity, and to further the understanding of underlying assumptions and values that go into these decisions.

## A Brief Account of the History of Severity as a Priority Setting Criterion on the Scandinavian Peninsula

Historically, public health services developed largely in parallel in Norway and Sweden. A comprehensive welfare state was introduced in the period following the Second World War. Both countries have comprehensive publicly funded health care systems. The total health expenditure as a percentage of GDP has grown steadily from about 1% in 1945 to around 10% today. Both nations recognised an urgent need to ration health care, and set up formal priority setting guidelines, starting in the early 1970s, after two decades of rapid growth of services and health budgets [99, 112].

It seems unfeasible to design a priority setting framework intended to curb unsustainable growth of health care expenditure without including some measure of cost-effectiveness. Both nations have de facto incorporated a *cost*-*effectiveness* principle as part of the backbone of priority setting guidelines. With regard to the measurement of effectiveness, over the last two decades the *quality*-*adjusted life year (QALY)* has become the canonical outcome measure for health technology assessment. The QALY is a preference based outcome measure integrating morbidity and mortality [118]. However, there seems to be a broad consensus among the public, politicians, health professionals and bureaucrats that cost-effectiveness alone does not capture all the salient attributes of illness and disease relevant to public priority setting, and that there are more to be said before fair and morally acceptable priority setting can be carried out. Particularly, the idea that an intervention or a treatment targeting patients with particularly severe conditions ought to be prioritised above treatment targeting patients with less severe health conditions, despite being less cost-efficient, has broad support in policy and among health care personnel and the citizens [29, 105]. Both Norway and Sweden have therefore included a *severity criterion.* How the severity criteria are being interpreted and implemented, however, are rather different.

### Severity in Norway

The first parliamentary committee concerned explicitly with priority setting in Norway, was the first Lønning commission in 1987 (Lønning I). As public health expenditure expanded rapidly from 1960 to 1980, previous commissions had already considered various aspects of rationing [61, 100], but Lønning I was the first comprehensive public inquiry into when and how rationing of health care could be carried out. In the resulting white paper (NOU)^2^ [59], a very thorough discussion of priority setting in health care is given, and several criteria are discussed. The commission’s mandate included analysing the de facto practices for priority setting in the Norwegian health care system, and—if found to be acceptable—to build upon these. To this end, Lønning I identified *degree of severity*, *egalitarianism*, *waiting time*, and *cost*-*effectiveness* as “relevant dimensions” for priority setting.

It might seem odd to identify *waiting time* as a criterion of priority setting; systematically making patients wait for indicated treatment is probably better understood as a mechanism for addressing more urgent needs first, for securing equality of access, and to some extent to ensure that *expectant management* is attempted before more costly treatment options are employed. That is, waiting lists are best understood as a priority setting tool. The discussion by Lønning I of waiting time in the health services highlights that patient’s wait for different reasons, and that while delayed treatment might be unacceptable for some, it is a reasonable rationing mechanism for others [59, pp. 77–78]. Lønning I also finds that egalitarianism, phrased as universal and equitable access to sufficient health care for the attainment of one’s “health potential” should remain an important principle of Norwegian health services. Nevertheless, it is severity that the commission considers the paramount parameter for priority setting, and severity is consistently linked with the concepts of *need*, *urgency*, *(risk of) death*, and *suffering*. Cost-effectiveness is also considered important in Lønning I, but, it seems, subordinate to the severity criterion.

Lønning II appeared in 1997 [60], and was the second parliamentary committee on general priority setting criteria^3^ for the Norwegian health services. The mandate for Lønning II explicitly called for an operationalisation of the principles from Lønning I, including the aim of making them applicable also to individual patients.

It is clear that Lønning II considers that integrating cost-effectiveness and the severity criterion is inherently threatened by the conflict between consequentialist and deontological ethics. The resolution of this dilemma is not carried out, and Lønning II recommends rather vaguely that human dignity must be the loadstar of priority setting, recommending that the different medical specialities must deliberate and decide how exactly severity is applied to their patients. Equity in health was considered to be subsumed by these principles [88].

In 2014 the Norheim commission completed the third NOU on priority setting—*Transparent and fair* [74]. This commission’s work upheld the focus on cost-effectiveness, but suggested a radical re-interpretation of severity and health equity. The *health loss* criterion—building on lifetime-prioritarianism—was proposed as sufficient for dealing with the severity issue [70, 72, 81, 82]. The ensuing debate was dismissive [7, 35, 52, 69, 71, 75, 80], and a second commission, led by Jon Magnussen, was appointed to reassess the severity criterion. The Magnussen report was submitted in 2015 [62], in which *absolute shortfall* was proposed as a better alternative to Norheim’s health loss criterion. Under the health loss criterion, a life-time prioritarian account is employed [82]. A condition’s severity is quantified by the number of life-time QALYs—considering both the past and the future—that an individual stand to lose, relative to a norm of 80 QALYs. This results in a priority setting criterion with a strong redistributive component. The absolute shortfall criterion differs in two important ways: it does not consider the past, but only considers the expected future QALY loss. Secondly, it does not assume a norm of 80 QALYs, but instead computes the gap to the age-specific expected number of remaining QALYs for an individual in good health. The absolute shortfall thus retains a redistributive aspect, but somewhat less pronounced, and no longer retrospectively. Both have in common that the QALY is the sole numeraire for severity.

An important feature of the Norheim-Magnussen debate is that, despite acknowledging that severity is ambiguous and multifaceted, the impact they had on official documents and priority setting debate was to highlight QALY-based measures of severity as the standard and to relegate other concerns to the periphery of the debate [77, 90]. Very simplified, we could say that Norheim’s health loss integrated severity and inequity and that Magnussen’s absolute shortfall fix to this (perceived) problem, largely deposed non-QALY concerns from consideration.

In 2018, another commission—the Blankholm commission—submitted yet another NOU: *The most important first* (*Det viktigste først)* [9]. This NOU discusses priority setting for municipal services, and largely concurs with Magnussen’s report on the issue of severity. However, an important deviation is implied by questioning the applicability of the QALY for non-specialist services—epitomised by the term *mastery* as an independent goal for treatment. The Norwegian term employed—‘*mestring’—*is perhaps better translated as *coping*, although *coping* here does not quite capture the full connotations of the Norwegian ‘mestring’, which suggests getting ‘on top of’ (e.g. capability to meet social roles, conduct activities of daily life, and find meaning and dignity.) rather than merely ‘getting by’. ‘Self-management’ is also a candidate term. Secondly, the Blankholm commission insists that a notion of *basic needs* should be defined and take precedence over severity (and equity) concerns in a priority setting context. The report goes as far as stating that covering these—still to be defined—basic needs should *not* be subject to cost-effectiveness constraints.

### Severity in Sweden

In Sweden a parliamentary committee on priority setting in health-care was established in 1992, resulting in a white paper in 1995 and in legislation in 1997 proposing three principles for priority setting: *The Human Dignity Principle*, the *Need*-*Solidarity Principle*, and the *Cost*-*Effectiveness Principle* [28, 87, 104, 105].

*The Human Dignity Principle* is the overriding ethical principle and addresses personal characteristics and functions in society that should *not* impact on priority setting (and therefore should not impact the assessment of severity), e.g., talent, social position, responsibility, income, chronological age, and gender. The concept of ‘need’ in the Needs-Solidarity principle is defined in terms of providing more resources to patients with the more severe diseases, but also worse quality of life, seemingly making a distinction between severe disease and bad quality of life. It is claimed that the care of severe disease and substantial quality of life decline should be prioritised before mild conditions, even if the former care is substantially less cost effective. Later in the white paper it is argued that a patient’s need is likely to depend both on the severity of the disease but also the duration of disease—seemingly making a distinction between current severity and duration.

It is claimed that a disease can vary in severity over time even for patients with chronic ill health conditions. It is also claimed that severity can be judged according to the suffering of the patient, the medical prognosis, the disability, and the existential despair experienced. In a later operationalisation by several central actors in the Swedish health-care system of the needs-solidarity principle, all of these different considerations were in some way subsumed under the concept of severity of a condition [85].

Moreover, severity has come to play an essential role in determining which treatments are subsidised by the Swedish pharmaceutical and benefits system (TLV), i.e. that which society pays for rather than the patient herself. More specifically, different thresholds of cost-effectiveness are considered acceptable depending on the degree of severity. For example, during the last few years, decisions indicate that a threshold of 1,000,000 SEK/QALY is accepted for conditions considered to have the highest degree of severity, 750,000 SEK/QALY for severe conditions, 500,000 SEK for conditions of moderate degree, and a similar decrement to the lowest degree. At present there are, however, different approaches to assessing severity in the Swedish health-care system, causing potential inconsistencies. Nonetheless, the policy of TLV is clearly different from the absolute shortfall policy of Norway: in Sweden severity is not measured using shortfall in terms of QALYs. At least not consistently. The Human Dignity Principle and its ban on taking chronological age into account has generally been interpreted as an obstacle to adopting an absolute shortfall approach to severity.

## Remaining Questions About Severity

There are numerous accounts of severity, as demonstrated by our overview of the Norwegian and Swedish discourses. A need to further clarify which basic moral considerations that should be involved is needed. However, no matter how important such activities are, deciding upon overarching considerations is not sufficient. To provide guidance, such considerations need to be specific, enabling them to be implemented in real world priority setting situations. Clarity regarding normative basis, as well as precision regarding practical implications, are two desirable traits of an account of severity. In order to achieve that, a set of questions about severity needs to be addressed.

The two most fundamental questions that need to be answered are: what is severity and why does it matter? Of course, it is difficult to see how the first answer could be answered without at least a tentative answer to the second one. However, the first question is essentially about what aspects or factors that should be included in an account of severity while the second refers to the feasibility and desirability of comparing different states of severity. Exploring these two main questions leads us to a number of sub-questions (see Table 1). Let us elaborate.

**Table 1:** Questions regarding severity

What is severity?	Why does severity matter?
Is severity dependent on individual desires?	Is severity an absolute or a relative concept?
What is the relationship between (subjective) well-being and severity?	Can severity be aggregated?
How should severity be assessed when patients suffer from more than one condition?	How does severity relate to considerations of equality?
How should severity be viewed from a temporal perspective?	How does severity relate to urgency and need?
What is the relationship between age and severity?	
Is death an independent dimension of severity?	
Is severity related to an individual’s social context?	
What is the relationship between severity and prevention?	

## Question I About Severity: What is Severity?

Let us start with the first question: what is severity? Perhaps the most important thing to determine when answering this question is which aspects should be taken into account when determining severity. As we have already seen from the Swedish and Norwegian cases, several suggestions exist for how to do this.

The Swedish white paper suggested that the suffering of the patient, the medical prognosis, the disability, and the existential despair experienced were all part of determining the level of severity [28]. At least, it was made clear that both health and quality of life mattered. Of course, whenever suggesting that more than one factor determines something, it is desirable to suggest the relative weight of these factors. Moreover, if one is referring to something as contested as quality of life and health, these concepts need to be understood unequivocally.

In Norway, a more well-defined but narrow understanding of the aspects determining severity has been adopted. In brief, the idea has been to understand severity and effect in the same terms: as quality adjusted life years (see above). Severity is quantified by some standard health economic measure, such as the preferences people express towards different health states as formalised by, for example, an EQ-5D questionnaire. Such measurements are usually considered to capture some notion of health-related quality of life [49]. But any such notion necessarily takes a stand on what is ultimately important for an individual, i.e. quality of life, and no such idea is uncontroversial [46]. Furthermore, the Norwegian take on severity presuppose that advantages (effect) and disadvantages (severity) should be understood in the same terms, which is by no means self-evident.

### Is Severity Dependent on Individual Desires?

One issue is whether the severity of a condition may depend on individual desires or preferences, or if severity is something which applies to a medical condition independent of the perceptions of the patient’s attitudes towards her condition. For example, the severity of infertility seems to depend on whether there is a desire for having children or not; if the individual has no desire of having biologically related children whatsoever, it is highly unlikely that the individual in question will perceive the condition of infertility as severe and unclear why anyone else should consider it so. It seems plausible to consider desires relevant for determining severity, at least sometimes and to some degree. But: when and how much? Moreover, will severity also depend on the strength of such a desire? This takes us to the general point about the relationship between subjective well-being and severity [42, 97].

### What is the Relationship Between (Subjective) Well-Being and Severity?

The seminal papers *Subjective well*-*being* and *The Satisfaction with Life Scale* by Ed Diener et al. have spawned a large literature with branches in psychology, philosophy, and economics. An important question for an account of severity for priority setting purposes is if and how it should incorporate subjective well-being [11, 23–26, 31, 78, 94, 114, 117]. We believe that a general sentiment among priority setting scholars is that without a clear idea of the problems arising from managing the gap between ‘objective’ health and ‘subjective’ well-being, policies risk coming into conflict with ethical ideals. For example, placing too much emphasis on subjective evaluation of one’s own health may discriminate against those who adapt well, or are endowed with a naturally positive disposition, while ‘objective measures of health’ may not measure what people in fact care about. Of course, such problems also concern the use of any preference based measure (e.g. QALYs).

### Should it Be the Disease or the Condition of the Patient that is Assessed?

In the Swedish context, there has been a shift from talking in terms of severity of disease towards discussing severity of ill health condition. The Swedish 1996 white paper about priorities in Sweden, stated that “[t]he diagnosis or disease is not important, the decisive issue is instead the condition at every particular time of need of health care” [87]. However, in the international discussion, both concepts are used. If the severity of disease assesses the disease regardless of treatment status, and the severity of a condition is sensitive to whether the condition has been treated or not, this may have a major impact on priority setting decisions, e.g. in situations where there is ample access to treatments reducing the severity of the disease [98].

### How Should Severity be Assessed When Patients Suffer from More than One Condition?

Normally, when severity is applied as a priority setting criterion in the Swedish or Norwegian systems, a specific condition is assessed (for instance, chronic heart failure with a specific NYHA level, prostate cancer at a specific stage etc.) in relation to a potential treatment targeting that condition (so-called condition-treatment pairs). This approach means that when severity is taken into account as a criterion for priority setting it is employed as the extent to which patients are badly off with respect to the condition that is targeted by the treatment (condition-specific severity). While there may be several practical reasons for this approach it is not clear whether it accounts for the morally relevant sense of being worse off. One alternative is to assess severity as the extent to which the patient is worse off when all of the patient’s conditions are considered [41]. However, this approach has a number of practical difficulties and it remains to be seen how plausible this approach is when related to other relevant criteria for priority setting. Moreover, some treatments target side-effects of a disease or treatment, for example managing nausea following chemotherapy. Does this mean that only the severity of nausea should be assessed or that the severity of the underlying cause of the nausea (in this case cancer) should also be considered? Or, if the patient suffers from both cancer and diabetes type II—should both conditions be taken into account in the assessment of severity—even if treatment only targets one of these conditions?

### How Should Severity be Viewed from a Temporal Perspective?

In a priority setting context, it is essential to have a clear idea of how a severity criterion applies to a condition over the course of treatment. Many conditions lead to prolonged treatment paths involving several treatment options at different times. It is important to establish whether the severity of a condition is subject to reassessment throughout the course of the disease. Another aspect of this is that if the severity of a condition approach is used, then treatment will affect the severity of the condition [98]. This implies that the order in which treatment are introduced might affect the actual prioritisation. For example, say we have two treatments *A* and *B* where *A* has a moderate effect but only has an acceptable level of cost-effectiveness if targeting a highly severe condition and *B* has a moderate effect but is acceptable even if targeting mildly severe conditions. If we consider a patient with a very severe disease, if the patient is given *B* first, the patient will not get access to *A*, since that is no longer acceptable given that the patient’s severity is now reduced. If, on the other hand, the patient is first given *A*, both treatments would be acceptable.

Another manifestation of how a temporal dimension of severity makes a difference is contained in the discussion of *health loss* versus *absolute shortfall* above: are certain *life*-*times* ‘severe’, or are certain *futures* ‘severe’. The latter view is forward-looking, and would not take into account for how long one has already suffered from a condition when determining severity. The first view is explicitly backward-looking, and holds that the past should be considered relevant for priority setting. Life-time prioritarianism, proposed by Norheim et al. in Norway, does accommodate the past [74, 81, 82]. Presently, both the Norwegian—after Magnussen’s report [62]—and the Swedish policy seem to reject the backward-looking view.

### What is the Relationship Between Age and Severity?

The problems related to age when using utility-like measures for health outcomes (QALY) and burden of disease (DALY) are well-known. As discussed above, there have been concerns that the hitherto proposed severity criteria in Norway and Sweden discriminate against the elderly. It is perhaps fair to say that any severity criterion that implicitly incorporates a shortfall of QALYs (or similar measures) must, to some extent, give lower priority to predominantly elderly patients as long as human longevity remains naturally bounded above. The question, then, is if this age-discrimination is a necessary consequence of those accounts of severity that is ethically defensible and which the public is willing to accept as legitimate. Moreover, should it be viewed as a case of *age*-*discrimination* (given that the chosen definition disadvantages those of advanced age)? Alternatively, severity must be described independently of QALY outcomes, in a manner that does not favour treatments that target younger patient groups [3, 12, 56, 66, 72, 81, 89, 118].

### Is Death an Independent Dimension of Severity?

Related to the former question is the question about severity and end-of-life concerns. There is a rich literature on priority setting in conjunction with end-of-life treatment, which suggests that many consider imminent death as an especially severe condition. However, it is also possible that the two issues—demise and severe conditions—have been inappropriately conflated. It is a mere observation that many conditions that lead to an early death are characterised as severe, but it is equally clear that there are non-fatal severe conditions. Furthermore, there is empirical evidence to the effect that the general public evaluates health differently when the prospect of death is salient, which indicates that death and severity are closely related, but not interchangeable [4, 5, 13, 17, 43, 63, 84, 101, 106–109, 125].

### Is Severity Related to an Individual’s Social Context?

Is the social context of the individual a dimension of severity? In the early years of the GBD’s DALY measure, explicit modelling of social context was included. If a caregiver suffers from a condition, his family or people close to him will most likely be affected in different ways. For example, the (now abandoned) age-weighting of the DALY attributed greater weight to illness and dysfunction during the reproductive years. Normally, in health-care we find that there is some form of obligations on health care providers also towards family or significant others. Are these obligations an indication for taking into account the effect on significant others’ well-being when assessing the severity of a patients’ condition, or vice versa, how the well-being of significant others affect the patient? Is it worse if the health condition also substantially affects other people, as is the case with e.g., dementia, psychiatric disease, or with young children? Conversely, is the medical condition less severe for the patient, when care and support from family and significant others are in place? In particular, if a shift towards more holistic approaches to well-being is advocated, these are important issues [1, 123]. Both the Swedish and the Norwegian policies seem to reject social context: the Swedish by explicitly rejecting this in its Human Dignity Principle, and the Norwegian by its general lack of focus on non-QALY measurable dimensions. However, the focus on the slightly indeterminate ‘mastery’ from the recent Blankholm NOU might change this in the coming years.

### What is the Relationship Between Severity and Prevention?

Can a non-severe condition be urgent because it will, in a near or more prolonged perspective, become severe untreated? A slight wound from a rabies infected dog is severe in the sense that if post-exposure prophylaxis is not administered, a fatal outcome is almost certain. Nevertheless, a dog-bite is often considered relatively minor in itself. Conversely, a condition leading to slight, but permanent, dysfunction could reasonably be considered urgent if expedient treatment could restore full function; severe, however, seems an unfit label. Or is the condition already inherently severe if urgent measures are needed?

How does the interplay between need, urgency and severity affect how we think about preventive measures, like vaccination programs, in priority setting contexts? In the preventive situation, we are treating a group in order for a small number of potential patients to have a benefit. When assessing severity, should the level of risk of developing the targeted condition be taken into account? In the Swedish context, the severity criterion has been recommended for use in assessment of preventive programmes by assessing the severity of the targeted condition once developed, and then lowering the severity somewhat given the level of risk to develop this condition [85]. In the Norwegian context, the severity of the condition in a preventive situation focuses on those individuals who will benefit from prevention, i.e., the severity of the condition avoided.

## Question II About Severity: Why Does Severity Matter?

Let us turn to the second general question: why is severity important to consider when prioritising in health care? There are at least two basic moral ideas that can buttress the importance of severity. First, we have the idea that being bad (or worse, or worst) off is morally important in itself and gives rise to special entitlements of receiving assistance from others. This kind of fundamental moral intuition (that can be labelled “the moral importance of evil”) is expressed in a multitude of specific ideas about just distribution. One is so-called *prioritarianism*, that says benefiting someone matters more the worse off that someone is (absolutely and not relative to others then). As mentioned, this view was presupposed in Norheim’s report [88]. Another version of the idea is *sufficientarianism*, saying that we are entitled to benefit when we are worse off than a pre-defined threshold of, say, health. Yet another is the Rawlsian difference principle, saying that we should make the worst off as well off as possible [48].

The other basic moral idea that may serve as a rationale for caring about severity is (comparative) egalitarianism, e.g. the idea that it is bad for oneself to be worse off than someone else, at least if it is of no fault of one’s own (so-called *telic* egalitarianism) [48]. That is to say, inequalities are intrinsically bad. This idea seems to be suggested in both the Norwegian and Swedish policies, but it is unclear what the connection is between egalitarian concerns and severity—see below. One suggestion is that severity matters because having a severe condition usually means that one is worse off than others and that we then have a moral reason to remedy this from the point of view of egalitarianism.

### Is Severity an Absolute or a Relative Concept?

A condition or a disease, it seems, is always severe in relation to something *non*-*severe*, say a normal, healthy state of existence. But apart from that, should the degree of severity be determined only (or foremost) in relation to how badly off one is in relation to others or should it be determined on a person-independent scale that in principle could be applied to any isolated individual (or condition)? That is, should “worse-offness” (so to speak) be considered relative to other individuals or in an absolute sense (in terms of how badly off one is regardless of others’ well-being)? The answer to this question turns on the answer to what the ultimate rationale of severity is, where the relative account seems to be the one favoured by egalitarians and the absolute account by those who view severity as founded on some idea about the moral weight of evil [43].

### Can Severity be Aggregated?

One question that an account of severity needs to address is the extent to which, one should consider severity as amenable to interpersonal aggregation. For instance, let us assume that we are considering distributing health care resources to one of two treatments to two separate groups of patients, *A* and *B*. Either, we can benefit *A* or *B*, but not both. Let us assume that those in group *A* suffer from a condition that is more severe than in group *B* and, consequently, that those in group B suffer from a less severe condition, but a condition that still is severe (or so we assume). Let us furthermore assume that the number of individuals in group *A* is smaller than in group *B*. Should we account for severity in such a way that it allows us to add up ‘severities’ in the respective groups so that the ‘total amount of severity’ in group *B* outweighs the total amount of severity in group *A* (even though all individuals in group *A* have more severe conditions than all individuals in group *B*)? In other words, should we consider severity as something that we can aggregate or not?

The answer to this question must hinge on the rationale behind caring about severity, i.e., the answer to the question: why does severity matter? If the answer is of a prioritarian kind, the answer seems to be *yes*, since prioritarianism allows all sorts of value comparison as long as weights are attached on being worse off [48]. On the other hand, if the answer is of a Rawlsian kind, the answer seems to be *no*, since the difference principle gives absolute priority to the worst off, regardless of the size of benefits to others [54]. This also seem to resonate with an account of severity which considers not having basic needs met—as alluded to by the Blankholm NOU—as severe; meeting basic needs of even one person could then compromise the needs of a myriad others. In fact, such an account seems to induce a non-Archimedean priority-ordering, where interventions targeting basic needs are *incommensurably* more important than others [37, 108].

### How do Severity and Equity Relate?

In particular, the Norwegian debate has been influenced strongly by prioritarianism though Norheim’s NOU, and its ethical heritage [6, 10, 20, 36, 53, 73, 74, 81, 82, 86, 111]. Very briefly, prioritarianism can be understood as a form of utilitarianism that incorporates some idea of equity, and is a normative and prescriptive ethical stance: severity *is—*at least in some measure—deprivation in relation to some more absolute threshold. If on the other hand, the normative background theory is some version of egalitarianism, severity is assessed relative to how other people are situated on the severity scale.

### How Does Severity Relate to Urgency and Need?

How does the notion of severity relate to other central concepts such as *need* and *urgency*? Some writers believe that need should be interpreted as either severity or capacity to benefit. It has also been suggested that a more plausible interpretation of a person’s needs-based claim on health care resources is dependent on both these aspects, namely the extent to which one is badly off as well as to what extent one can benefit from an intervention [18, 40]. According to this interpretation, severity is an attribute of one’s needs. However, a delicate issue for this proposal is to work out a more precise answer to how needs-based claims should relate to the trade-off between these two aspects.

## Conclusion

In this article we have identified several crucial shortcomings of the current debate over the use of severity criteria in priority setting. Our list of questions should be seen as a work-in-progress and can be used in several ways. The obvious one is as an inspiration for researchers within the field of priority setting and resource allocation. It can also serve as a point of departure for discussions among policymakers and also as a first step in research on priority setting processes, where different stakeholders will be engaged in creating consensus on the most urgent issues to pay attention to in order to facilitate policy development, and more importantly, facilitate implementation.

## Footnotes

1 Both Norway and Sweden are generally acknowledged to have fairly extensive *universal health care (UHC)* systems, although particularly illegal immigrants are known to suffer from poor access to basic health care [68, 79].

2 In Norway, a white paper summing up the reasoning and conclusions of a governmental committee deliberating on a topic is referred toa s an NOU (Norsk Offentlig Utredning). The equivalent term for Swedish white papers is SOU (Statens offentliga utredningar).

3 Note that two other commissions—Grundt (remuneration for pharmacological expenses) and Steine (hospital management principles)—handed over NOUs in 1997, and these commissions’ reports display substantial overlap in arguments and conclusions [38, 110]. For example, all three reports reproduce the same figure for an illustration of what has later become known as the “health gap”—the discrepancy between the technical and the financial constraints on treatment options.
